# Lady in Red: A Fatal Case of Drug Reaction With Eosinophilia and Systemic Symptoms

**DOI:** 10.7759/cureus.82235

**Published:** 2025-04-14

**Authors:** Reese M Cargioli, Brian Nguyen, Hytham Rashid, Ben Drake, Stephen Fletcher

**Affiliations:** 1 Department of Internal Medicine, University of Miami, Coral Gables, USA; 2 Department of Graduate Medical Education, University of Houston College of Medicine, Houston, USA; 3 Department of Internal Medicine, University of Houston/HCA Houston Healthcare, Houston, USA; 4 Department of Internal Medicine, Merit Health Wesley, Hattiesburg, USA; 5 Department of Internal Medicine, East Alabama Internal Medicine Associates, Opelika, USA

**Keywords:** adult hospital medicine, allergy/immunology, dermatology, dress, dress reaction with eosinophilia and systemic symptoms, drug rash, drug reaction, drug reaction with eosinophilia and systemic symptoms (dress) syndrome, persistent diarrhea

## Abstract

Drug reaction with eosinophilia and systemic symptoms (DRESS) is a rare, life-threatening drug-induced hypersensitivity reaction. Here, we present the case of a 72-year-old female patient who developed DRESS after starting allopurinol, a well-known causative agent. The patient initially presented to Urgent Care with rash and diarrhea where she was prescribed methylprednisolone dose pack, diphenhydramine as needed, and metronidazole for possible colitis. She had multiple outpatient clinic and emergency department visits for follow-up of fluctuating symptoms and a persistently elevated WBC count despite multiple courses of antibiotics for presumed colitis. After a prolonged clinical course, she was diagnosed with DRESS and admitted for high-dose glucocorticoids. Unfortunately, her condition deteriorated after transfer to the intensive care unit, and she passed away. This case highlights the importance of early consideration, diagnosis, and treatment of DRESS to avoid potentially fatal outcomes.

## Introduction

Drug reaction with eosinophilia and systemic symptoms (DRESS) is a rare, life-threatening drug-induced hypersensitivity reaction. It is usually triggered by medication, with allopurinol being one of the most implicated medications [1,2]. As it accounts for only a small percentage of drug reactions, DRESS poses a particular diagnostic challenge due to its variable presentation and delayed onset of symptoms [3]. Early recognition, discontinuation of offending agents, and initiation of glucocorticoid therapy is vital to the management of this condition as a delay in diagnosis and treatment can lead to severe complications including multiorgan failure and death.

The pathophysiology of DRESS is variable and includes T-cell-mediated immune responses along with other generic factors associated with human leukocyte antigen (HLA) [4,5]. DRESS often presents with delayed symptoms, typically two to eight weeks after the initiation of the offending agent, further complicating early detection [6]. In addition to this delayed response, symptoms can present across a wide range and often overlap with other infectious, autoimmune, and drug-induced conditions [7]. The Registry of Severe Cutaneous Adverse Reactions (RegiSCAR) scoring system incorporates laboratory and clinical criteria to assess the likelihood of DRESS in patients presenting with this rare syndrome [1].

This case report illustrates the diagnostic complexities, clinical course, and fatal outcome in a 72-year-old female patient who developed DRESS following the initiation of allopurinol for gout. It highlights the importance of maintaining a high index of suspicion for DRESS in patients with multi-organ dysfunction in the setting of a common offending agent. Also, it illustrates the multiorgan involvement and utility of RegiSCAR scoring in patients presenting with DRESS.

## Case presentation

A 72-year-old woman with a history of hypertension treated with lisinopril and carvedilol, type 2 diabetes mellitus treated with metformin, and newly diagnosed gout treated with allopurinol initially presented to Urgent Care for abdominal rash and diarrhea that started approximately 10 days prior. She reported that one week after starting allopurinol, she developed erythematous patches on her stomach followed by non-bloody diarrhea. She was diagnosed with possible colitis and was prescribed metronidazole, methylprednisolone dose pack, and diphenhydramine and was advised outpatient follow-up.

The patient followed up with a primary care provider (PCP) and was found to have worsening rash and persistent diarrhea, but was otherwise afebrile and hemodynamically stable. She was instructed to continue metronidazole for presumed colitis. She returned two days later with a worsening rash and unresolved non-bloody diarrhea. Due to persistent diarrhea and concern for colitis, metronidazole was discontinued and levofloxacin was started. She was advised to continue diphenhydramine as needed for the rash. The patient’s son reported at this visit that her symptoms began one week after starting allopurinol and the patient was advised to discontinue it. A complete blood count (CBC) showed a WBC count of 19,000 cells/uL (normal: 5000-12,000), which was attributed to the recent use of methylprednisolone dose pack.

Two days later, she returned to her PCP with still worsening symptoms. A repeat WBC count remained elevated at 18,000 cells/uL. A computed tomography (CT) scan of the abdomen was non-contributory demonstrating no acute pathology. She returned five days later with improving rash, but new fatigue, fever, nausea, and vomiting. The patient was referred to the ED for further evaluation due to presumed failure of outpatient treatment for colitis.

On arrival to the ED, she was hemodynamically stable and afebrile, but appeared confused with diffusely tender abdomen. Her CBC demonstrated leukocytosis with the WBC count at 20,000 cells/uL, with eosinophilia (22%), which remained elevated throughout her hospitalization. She was started on intravenous (IV) fluids piperacillin/tazobactam for suspected UTI. Repeat CT of the abdomen remained noncontributory. Neurology and Gastroenterology (GI) were consulted and both advised outpatient follow-up. Blood cultures from the initial admission remained negative and the patient was discharged approximately two weeks after admission.

**Figure 1:**
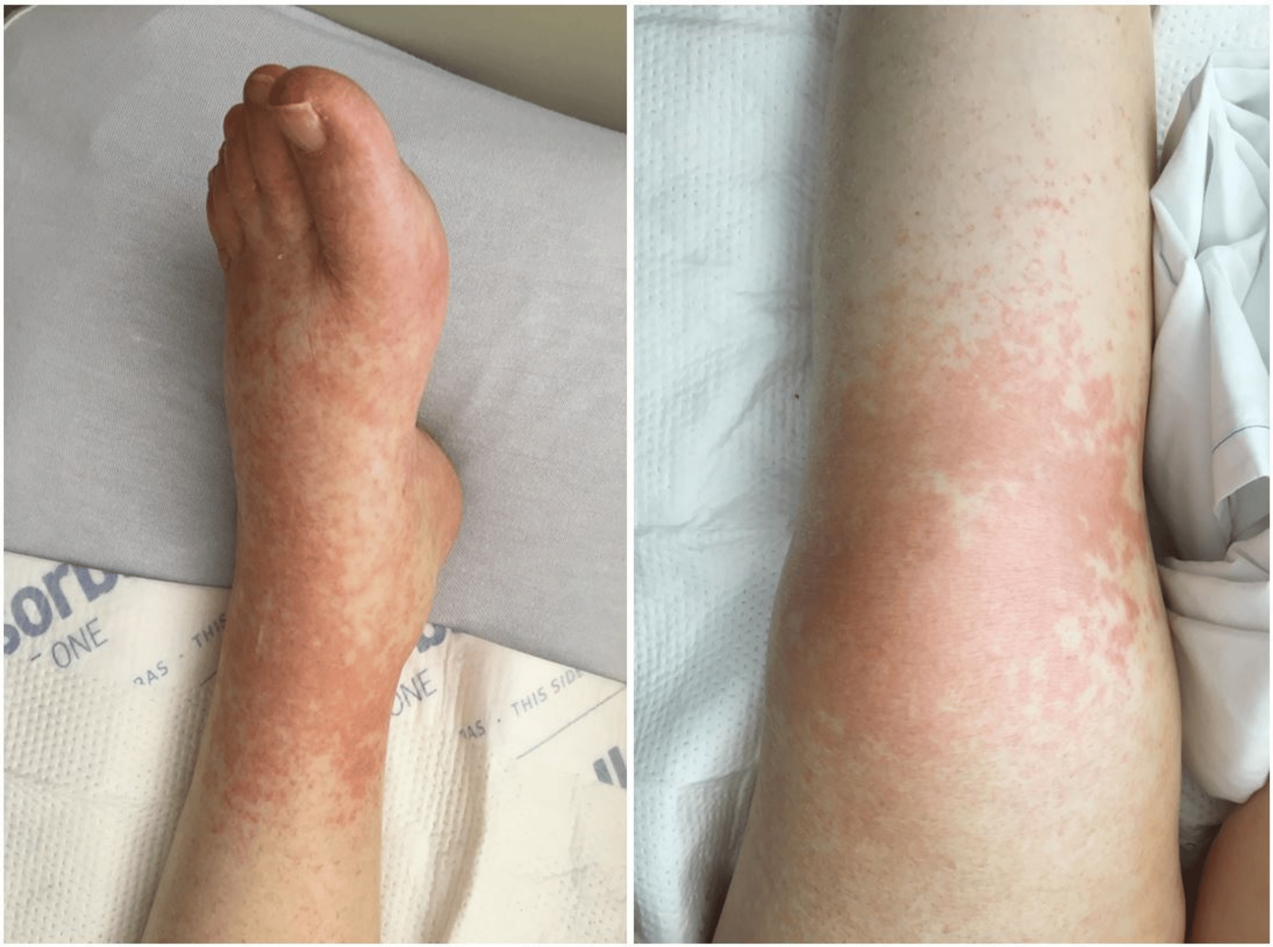
Left lower extremity exhibiting erythematous, maculopapular lesions

She returned to the ED three days after discharge with recurrent rash and diarrhea. She again was hemodynamically stable and afebrile, but her abdominal rash was non-desquamating and involved her lower limbs (Figure 1). CBC showed resolution of her eosinophilia, but liver function tests (LFTs) were more than three times the upper limit of normal with normal lipase levels. Due to concerns for DRESS, she was started on high-dose systemic glucocorticoid therapy and admitted for close monitoring. A skin biopsy performed showed leukocytoclastic predominance. On day 3 of hospitalization, her condition worsened despite glucocorticoids. She required intubation and was transferred to the intensive care unit (ICU) due to cardiopulmonary arrest. Despite IV fluid resuscitation and multiple vasopressors, she remained hypotensive and expired two days later.

## Discussion

Drug reaction with eosinophilia and systemic symptoms is a potentially life-threatening adverse drug reaction with a wide range of manifestations including fever, rash, lymphadenopathy, and multiorgan involvement with laboratory results often showing leukocytosis, eosinophilia, and abnormal kidney and liver function [1]. This case highlights the diagnostic challenges, importance of early diagnosis, and the poor outcomes associated with DRESS, especially in elderly patients with comorbidities. The patient’s clinical course underscores the importance of early recognition, discontinuation of offending medications, and prompt initiation of immunosuppressive therapy.

Allopurinol is a well-documented trigger for DRESS with a significant proportion of cases in the literature. One RegiSCAR study found that 18% of possible DRESS cases involved allopurinol. Other drugs considered to induce DRESS in this study included antiepileptic drugs (35%), sulfonamides (12%), and other antibiotics (13%) [1]. Another smaller case series of 38 patients found that 37% of patients had been exposed to allopurinol prior to their development of DRESS [2]. Furthermore, the delayed onset of symptoms of two to eight weeks following the initiation of allopurinol, as seen in our patient, is typical of DRESS [6]. No exact mechanism has been identified to cause DRESS, but there are several important factors believed to contribute to its development, particularly as it relates to allopurinol. A genetic predisposition in HLA has been shown to be a significant factor in allopurinol-associated DRESS in the Han Chinese population [4]. Additionally, the reactivation of Epstein-Barr virus (EBV), herpes human virus 6 (HHV-6), and HHV-7 leading to a T-lymphocyte-mediated inflammatory response has also been associated with DRESS due to allopurinol use [5].

DRESS demonstrates a particular diagnostic challenge due to its multiorgan involvement and variable presentations. In our case, the patient’s diarrhea was focused on and anchored to, despite failure to improve with multiple rounds of antibiotics and other associated symptoms including rash, eosinophilia, elevated liver enzymes, confusion/altered mental status, and negative infectious work-up. Gastrointestinal symptoms pose a particular challenge to the diagnosis of DRESS as they are rare, but well documented [2,8]. Liver involvement occurs in up to 70%-90% of cases, eosinophilia in approximately 30%, and morbilliform rash in 80% [3]. There is no pathognomonic finding for DRESS on skin biopsy and these findings may include perivascular inflammation, lymphocyte infiltration, and dermal edema [6]. The RegiSCAR score can be used to assess a patient’s likelihood of DRESS, and in our case, the patient symptoms led to a RegiSCAR score of 6 that correlates to a definite diagnosis of DRESS syndrome (Table 1).

**Table 1:** RegiSCAR scoring system for classifying DRESS DRESS: drug reaction with eosinophilia and systemic symptoms; RegiSCAR: Registry of Severe Cutaneous Adverse Reactions Table adapted under the open-access Creative Common CC BY 4.0 license from [9].

Item	Present	Absent	Our patient
Fever ≥38.5°C (101.3°F)	0	−1	0
Enlarged lymph nodes (>1 cm size, at least two sites)	1	0	0
Eosinophilia: ≥700 cells/mm^3^ or ≥10% (leucopenia)	1	0	0
Eosinophilia: ≥1500 cells/mm^3 ^or ≥20% (leucopenia)	2	0	2
Atypical lymphocytes	1	0	0
Rash ≥50% of body surface area	1	0	1
Rash suggestive (≥2 of facial edema, purpura, infiltration, desquamation)	1	0	0
Skin biopsy suggesting alternative diagnosis	−1	0	0
Organ involvement: one	1	0	0
Organ involvement: two or more	2	0	2
Disease duration >15 days	0	−2	0
Investigation for ≥3 alternative causes (blood cultures, antinuclear antibody, serology hepatitis viruses, Mycoplasma, Chlamydia) done and negative	1	0	1

The initial management of DRESS includes discontinuing suspected causative medications, and initiation of systemic glucocorticoids. Prompt administration of high-dose corticosteroids is the cornerstone of treatment for severe DRESS and has been shown to reduce mortality and morbidity [7]. For mild cases of DRESS, watchful waiting may be appropriate. For patients allergic to or not responding to systemic corticosteroids, cyclosporine, intravenous immunoglobulin (IVIG), and plasmapheresis are alternative therapies [10]. Our patient’s rapid progression to multiorgan failure and death in her second admission, despite the initiation of glucocorticoids, illustrates the unpredictable and fulminant course that DRESS can take, especially in high-risk elderly populations.

## Conclusions

This case demonstrated the diagnostic and therapeutic challenges associated with the identification and treatment of DRESS. Despite discontinuation of allopurinol and initiation of glucocorticoids, the patient’s condition rapidly deteriorated, culminating in multiorgan failure and death. This outcome emphasizes the importance of early recognition and treatment of DRESS and potential fatal consequences of a missed diagnosis. Clinicians should maintain a high index of suspicion for DRESS in patients presenting with rash, eosinophilia, and systemic symptoms such as liver or kidney damage, especially in the context of a recent exposure to high-risk medications like allopurinol. Additional research is needed to better understand the pathophysiology of DRESS and to develop more effective diagnostic and treatment strategies to reduce morbidity and mortality associated with this potentially fatal adverse drug reaction.
